# Short-Term Effect of Continuous Subcutaneous Insulin Infusion and Multiple Daily Injection in Perioperative Patients with Type 2 Diabetes Mellitus

**DOI:** 10.1155/2023/8542262

**Published:** 2023-06-23

**Authors:** Wei Song, Jianxuan Wen, Jinming Zhang, Meng Luo, Yue Hu, Yu Zhang, Guanjie Fan, Ling Zhao

**Affiliations:** ^1^The Second Affiliated Hospital of Guangzhou, University of Chinese Medicine, Guangzhou, 510210 Guangdong Province, China; ^2^Department of Endocrinology, Huizhou Hospital of Guangzhou, University of Chinese Medicine, Huizhou, 516000 Guangdong Province, China; ^3^Second Clinical College of Guangzhou, University of Chinese Medicine, Guangzhou, 510120 Guangdong Province, China

## Abstract

**Background:**

Hyperglycemia is common and difficult to control in perioperative patients with type 2 diabetes mellitus (T2DM), which impacts their prognosis after operation. Our study investigated the short-term effect of continuous subcutaneous insulin infusion (CSII) and multiple daily injection (MDI) in perioperative T2DM patients using the data envelopment analysis (DEA).

**Methods:**

T2DM patients (*n* = 639) who underwent surgeries in Guangdong Provincial Hospital of Traditional Chinese Medicine (2009.01-2017.12) were included. Insulin was provided to each patient during the study and separated into a CSII group (*n* = 369) and an MDI group (*n* = 270). DEA was performed to compare the therapeutic indexes and investigate the short-term effect of the CSII group and MDI group.

**Results:**

Scale efficiencies of the CSII group with CCR model and BCC model were better than that of the MDI group. Regarding slack variables, with higher surgical levels, the CSII group was closer to the ideal state than the MDI group, which indicated in improving the average fasting blood glucose (AFBG), antibiotic use days (AUD), preoperative blood glucose control time (PBGCT), first postoperative day fasting blood glucose (FPDFBG), and postoperative hospitalization days (PHD).

**Conclusion:**

CSII could effectively control blood glucose levels and shorten perioperative hospitalizing time for T2DM patients, indicating that CSII was beneficial in perioperative period and should be promoted clinically.

## 1. Introduction

Type 2 diabetes mellitus (T2DM) caused by abnormal blood sugar metabolism is a widely seen chronic disease that affects human health and quality of life. With living standard's improving and changes in living habits, the incidence rate has increased significantly [1]. In recent years, an increasing number of patients with T2DM have undergone surgery, but the hyperglycemic state associated with diabetes affects a patient's internal environment and physiological functions, enhances the likelihood of surgery and infection, and affects wound healing. Studies have pointed out that strict control of perioperative blood glucose in patients with T2DM can improve the effect of surgery and promote postoperative healing [2, 3]. Insulin therapy is the optimal treatment for perioperative patients with T2DM to control blood glucose [4], which includes continuous subcutaneous insulin infusion (CSII) and multiple daily injection (MDI). CSII is a new type of glucose control instrument in clinical practice in recent years, which can mimic the physiology of insulin secretion in patients and continuously pumps insulin for patients to stabilize blood glucose levels. It has been reported that CSII effectively alleviated hyperglycemia in patients who had a suboptimal response to therapy for T2DM, which is more effective than MDI [5]. However, there is no study to evaluate which of the two methods of CSII or MDI is more beneficial in health economics. Data envelopment analysis (DEA), first proposed by Wei and Charnes et al., is a method to systematically evaluate the relative effectiveness of decision-making units of the same type according to specific input/output indexes. It is one of the operation research methods in comprehensive evaluation methods [6, 7]. DEA can evaluate the relative efficiency of treatment schemes with single or multiple input indexes and multiple output indexes and select more effective schemes from them. At the same time, it can analyze the inputs and outputs of relatively inefficient schemes, which is conducive to further improvement of inefficient schemes, so that each decision unit can obtain the best benefits. In recent years, DEA has gradually been used to evaluate the prevention and treatment of different chronic diseases, such as diabetes. Therefore, this study retrospectively analyzed the perioperative patient with T2DM in Guangdong Provincial Hospital of Traditional Chinese Medicine in the last 7 years and utilized DEA to investigate the clinical efficacy and health economic benefits of CSII and MDI in treating the perioperative patient with T2DM during perioperative period.

## 2. Materials and Methods

### 2.1. General Information

The study included 639 patients with T2DM who had undergone surgery in our hospital from January 2009 to November 2017. According to the treatment plan, the patients were divided into a CSII group (*n* = 369) and an MDI group (*n* = 270).

#### 2.1.1. Inclusion Criteria

The inclusion criteria are as follows:

1. Patients that were diagnosed with T2DM with the criteria established by WHO in 1999
2. All patients that had indications for surgery and were ready for surgery
3. Patients that were treated with CSII or MDI

#### 2.1.2. Exclusion Criteria

The exclusion criteria are as follows:

1. Patients with severe endocrine system, nervous system, immune system, or mental system diseases
2. Patients with severe heart and lung dysfunction or severe liver and kidney dysfunction

### 2.2. Methods

Baseline comparisons were performed between the two groups using SPSS 17.0, and the data are expressed as mean ± SD. If the quantitative data were normally distributed and the variances were equal, the data were analyzed with *t*-tests. If not, the data were evaluated by rank sum test. Qualitative data were assessed with chi-square test. In data envelopment analysis, the input indicators of both groups were the total hospitalization cost and hospitalization days. The output indicators were the average fasting blood glucose (AFBG), antibiotic use days (AUD), preoperative blood glucose control time (PBGCT), first postoperative day fasting blood glucose (FPDFBG), and postoperative hospitalization days (PHD). Each included patient in the decision-making unit was analyzed using the Charnes, Cooper, and Rhodes (CCR) model and the Banker, Charnes, and Cooper (BCC) model. The efficiency values of each decision-making unit were calculated with DEAP 2.1 software. The average value for each group was taken, and the average efficiency values of the two groups were compared. Then, two groups of slack variables were analyzed and compared to draw conclusions.

## 3. Results

### 3.1. Comparison of Patients' Baseline Characteristics

No difference in sex, age, course of disease, or type of surgery between 2 groups was found. The HbA1c level in the CSII group was significantly higher than that of MDI group, suggesting that CSII group patients had higher blood glucose levels and worse condition than patients in the other group (Table 1).

### 3.2. Hospitalization Days and Hospitalization Costs

Results indicated that no difference in the length of hospitalization between the 2 groups was shown. The total hospitalization cost in the CSII group was significantly higher than that of the MDI group (Table 2). Note: by t-test, no difference in hospitalization days, but a significant difference in total cost between 2 groups was found. CNY: Chinese Yuan.

### 3.3. Data Envelopment Analysis Results

#### 3.3.1. Input-Output Indicators

A total of 639 patients were treated as independent decision-making units. The input indicators of both groups were the total hospitalization cost and hospitalization days. The output indicators were PBGCT, AFBG, FPDFBG, AUD, and PHD.

#### 3.3.2. Analysis of Efficiency Values in the Two Groups

Input indicators and output indicators were collected for 639 patients who completed the study, and these data were analyzed using DEAP software. The average efficiency of the two groups was taken as the overall efficiency value. The results (Table 3) show that the efficiency values in the CSII group with CCR/BCC model were better than those of the MDI group.

#### 3.3.3. Analysis of Slack Variables in the Two Groups

The slack variable indicates the gap between actual and ideal output. The smaller the slack variable, the closer it is to ideal state. According to Table 4, we can conclude that the CSII group was closer to the ideal state than the MDI group in terms of improving AFBG, FPDFBG, AUD, and PHD, while the MDI group was closer to the ideal state than the CSII group in terms of PBGCT and FPDFBG. Note: BG: blood sugar. FBG: fasting blood sugar.

### 3.4. Stratified Analysis

In China, surgery is allocated into 4 grades based on complexity, difficulty, and risk according to medical surgery grading management system by National Health Commission of the People's Republic of China (https://www.nhc.gov.cn/wjw/ywfw/201306/def185b8d52e48918cf7e12e43e956d6.shtml). Accordingly, surgeries of lower difficulty, simpler procedure, and lower risks are classified as grade I, while surgeries with the highest risks, the most complex procedure, and the greatest technical difficulty are classified as grade IV surgeries. The two groups of patients were stratified according to the above-mentioned four levels of surgery. The CSII group included 79 patients who underwent grade I surgery, 59 patients who underwent grade II surgery, 136 patients who underwent grade III surgery, and 95 patients who underwent grade IV surgery. The MDI group included 61 patients who underwent grade I surgery, 81 patients who underwent grade II surgery, 64 patients who underwent grade III surgery, and 64 patients who underwent grade IV surgery.

#### 3.4.1. Grade I Surgery

The comparison of patients undergoing grade I surgery between the two groups (Table 5) showed that the CSII group was closer to ideal state than the MDI group in terms of improving AFBG, FPDBG, AUD, and PHD, while the opposite result was found in terms of PBGCT and FPDFBG.

#### 3.4.2. Grade II Surgery

In terms of grade II surgery, the CSII group was closer to the ideal state in terms of improving PBGCT, AFBG, AUD, and PHD. However, the MDI group was better than the CSII group in terms of first postoperative day fasting blood glucose and first postoperative day average blood glucose (Table 6).

#### 3.4.3. Grade III Surgery

At the grade III surgery level, the CSII group was closer to the ideal state than the MDI group in terms of improving each output indicator (Table 7).

#### 3.4.4. Grade IV Surgery

At the grade IV surgery level, the results were the same as those for grade III surgery (Table 8).

## 4. Discussion

Diabetes mellitus is a common chronic disease induced by many factors, and it drastically lowers patients' quality of life. Once patients begin to suffer from this disease, it is necessary to take drugs for a long time to control it, and the course of diabetes mellitus is very long. Even in the treatment process, chronic complications or target organ damage caused by hyperglycemia, which is especially obvious in elderly diabetic patients, easily occurs [8]. Long-term hyperglycemia will cause high glucose toxicity, which may lead to the suppression of leukocytes in patients, may affect the function of leukocytes, may weaken the body's immunity, and may reduce the body's resistance to the disease. These are the main reasons why diabetes patients are prone to infection [9]. In regard to surgery, a blood glucose concentration that is too high will also affect the function of endothelial cells in patients' bodies and will affect the synthesis of collagen, which induces poor wound healing and prolongs incision healing time [10, 11]. At the same time, hospitalization due to hyperglycemia will significantly increase the incidence of complications and death risk in patients. It will significantly increase the infection risk in surgical patients and prolong the healing time of surgical wounds, resulting in longer hospital stays and increased hospitalization costs [2, 3, 12]. Therefore, timely detection and systematic management of hospitalized hyperglycemia are of great significance to endocrinology and nonendocrinology departments. CSII and MDI are two common methods of hypoglycemic therapy in clinical use. Although MDI is economical and convenient, it is difficult to simulate the 24-hour physiological insulin secretion curve in patients. Patients' blood sugar concentrations fluctuate greatly, and the effect of blood glucose control is poor because the insulin level between meals is relatively higher than the normal level, while the corresponding peak insulin level after meals is relatively lower [13]. The insulin pump known as CSII mainly adopts the form of a basal dose and bonus dose to input insulin, allowing for intensive insulin therapy, which can achieve continuous insulin microinput for 24 hours according to the body's normal insulin secretion in order to stably control blood sugar and reduce risks of hyperglycemia and hypoglycemia [14]. CSII is widely used in treating type 1 diabetes, and study has also shown that CSII has a significant advantage in T2DM patients with poor control of blood glucose [15]. In the perioperative period, the use of CSII is more advantageous, which can help patients to achieve the target blood glucose quickly and shorten the preoperative preparation time [16]. Studies have shown that CSII in diabetic patients is the best choice for cost effectiveness, although the amount of insulin is more and the cost is higher, but the cost-effectiveness ratio and incremental cost-effectiveness ratio are both smaller [17]. For now, there are few studies on the health economics of CSII and MDI during the perioperative period. Therefore, further research is needed. The evaluation methods of health economics mainly include analysis of cost-effectiveness, cost utility, cost benefit, and cost minimization [18]. However, each method has its own advantages and limitations. For example, cost-effectiveness analysis might be adopted to evaluate multiple schemes, but quality-adjusted life years of patients are difficult to be fixed. DEA can comprehensively evaluate the curative effect and economic benefit of multiple programs, especially for multiple outputs, which is more advantageous. The comprehensive evaluation of treatment programs by using DEA will also bring us a more economical, effective, and close to clinical practice. Compared with traditional analysis methods, DEA has the following advantages. First, while evaluating the relative effectiveness of the program, DEA does not emphasize the consistency and singleness of the intervention measures and only requires that all decision-making units be of the same type. Secondly, while evaluating the relative effectiveness of the plan, DEA can also analyze the input and output of each plan group and point out whether the input and output are optimized, which will help us to further improve the treatment plan continuously so as to better serve patients [19, 20]. For instance, DEA has been adopted [21] to evaluate the efficiency and performance of prevention and treatment for chronic diseases such as diabetes in the United States, and they proposed several intervention plans for the inefficient states to maintain and improve statewide health care services for the American population. Zhang et al. [22] applied DEA to conduct economic comprehensive evaluation on input and output indexes of metformin enteric-coated tablets combined sulfonylureas (group A), acarbose combined insulin glargine (group B), and premixed insulin (group C), respectively. Data indicated that input and output of the premixed insulin group were optimized, while the other two groups needed to be improved. Our data revealed that HbA1c levels of the CSII group were significantly higher than that of MDI, but the number of hospitalization days was not increased. The use of CSII may account for this result, suggesting that CSII has better efficiency for rapidly lowering blood sugar in order to shorten the hospitalization stay. The total treatment expense of the CSII group was higher, which may be relevant with higher blood glucose level in patients. Through data envelopment analysis, the efficiency values in the CSII group with CCR/BCC model were better than those of the MDI group. From the analysis results of slack variables, the higher the surgical level, the closer the CSII group was to ideal state compared to the MDI group in terms of improving PBGCT, AFBG, FPDFBG, AUD, and PHD, suggesting that the application of CSII in surgery may have better benefits. CSII did not bring better economic and health benefits in grade I and II surgeries. The perioperative patients who undergo grade III and above surgeries will be suggested to be treated with CSII, which is more beneficial to blood sugar control and shorten hospitalization time, but it may not reduce the total hospitalization cost of patients. Subcutaneous insulin injections are still the most commonly used method of insulin delivery, despite their limitations such as fear of needles, bleeding, and fat atrophy, as well as the inconvenience of injection [23, 24]. As a result, researchers have been exploring alternative routes of administration and their effects on treatment outcomes. Studies have mainly focused on administering insulin through the ocular, nasal, buccal, oral, and dermal routes. Ocular administration, while simple to perform and taking advantage of the ocular tissues' immunosensitivity, has low bioavailability and slow absorption rates that require higher doses or absorption agents, which can cause irritation of the eye [25]. Alternatively, oral administration through an ingestible self-directing millimeter drug delivery device, developed by Abramson et al., shows high acceptance and convenience for patients [26]. However, the efficacy is limited due to barriers such as physical, chemical, and biological factors, requiring high doses of insulin for effectiveness. Insulin has a significant impact on the body's immune system. It exerts both an anti-inflammatory response and enhances the function of immune cells, potentially playing a crucial role in immune cell differentiation and development. Recent findings suggest that insulin may also shift the immune response from innate to adaptive during prolonged immune activation [27]. While insulin appears to enhance some immune functions, studies have demonstrated that insulin may also be proinflammatory by opposing the function of anti-inflammatory Treg cells. Furthermore, sustained inflammatory injury may inhibit insulin release through apoptosis. However, insulin treatment can promote glucose uptake by immune cells, and milder doses of insulin may actually enhance immune function. The duration of insulin exposure and the timing of response measurements may also impact immune cell phenotype and immune response [28]. Tsai et al. [29] used a mouse model with a T cell-specific conditional Insr knockdown, finding that Tregs isolated from hyperinsulinemic obese mice had an elevated expression of IFN-*γ* and inhibited IL-10 release. Additionally, while TNF-*α* treatment of 30 min *β* cells reduced insulin secretion, extended 24 h exposure attenuated insulin release from cultured *β* cells.

## Figures and Tables

**Table 1:** Patients' baseline characteristics.

	Group	*N*	*X* ± *S*	*P*
Age	MDI group	270	62.91 ± 11.330	0.077
	CSII group	369	61.96 ± 13.555	
Course (months)	MDI group	270	159.80 ± 145.79	0.126
	CSII group	369	89.55 ± 97.38	
Weight	MDI group	270	61.942 ± 11.2950	0.3990
	CSII group	369	62.246 ± 11.9811	
HbA1c	MDI group	270	8.280 ± 2.0847	0.0001
	CSII group	369	10.579 ± 2.9034	

Note: by chi-square test, *P* > 0.05, no difference in sex between 2 groups.

**Table 2:** Comparison of hospitalization days and hospitalization costs.

	Group	*N*	*X* ± *S*	*P*
Hospitalization day (days)	MDI group	270	16.04 ± 9.22	0.996
	CSII group	369	16.79 ± 9.53	
Total hospitalization cost (CNY)	MDI group	270	27407.52 ± 18519.71	0.001
	CSII group	369	44407.12 ± 49029.92	

**Table 3:** Comparison of efficiency values.

Group	CCR model efficiency value (*θ*)	BCC model efficiency value (*θ*)
CSII group	0.762	0.820
MDI group	0.754	0.764
Overall efficiency value	0.704	0.689

**Table 4:** Analysis of slack variables in the two groups.

Group	Preoperative		Postoperative day 1		Days of antibiotic use	Postoperative hospitalization days
	BG control time	Mean FBG	FBG	Mean BG		
CSII group	0.615	0.571	1.764	1.135	0.446	0.154
MDI group	0.091	1.585	1.496	1.305	0.557	0.219
Total mean	0.286	0.918	1.216	1.337	0.999	0.231

**Table 5:** Analysis of slack variables in patients undergoing grade I surgery.

Group	Preoperative		Postoperative day 1		Days of antibiotic use	Postoperative hospitalization days
	BG control time	Mean FBG	FBG	Mean BG		
CSII group	0.112	0.504	0.329	0.678	0.010	0.128
MDI group	0.102	1.023	0.458	0.567	0.118	0.378
Total mean	0.100	0.638	0.328	0.704	0.020	0.286

**Table 6:** Analysis of slack variables in patients undergoing grade II surgery.

Group	Preoperative		Postoperative day 1		Days of antibiotic use	Postoperative hospitalization days
	BG control time	Mean FBG	FBG	Mean BG		
CSII group	0.107	0.706	0.289	0.493	0.001	0.118
MDI group	0.112	1.102	0.276	0.387	0.109	0.295
Total mean	0.108	0.879	0.213	0.504	0.033	0.260

**Table 7:** Analysis of slack variables in patients undergoing grade III surgery.

Group	Preoperative		Postoperative day 1		Days of antibiotic use	Postoperative hospitalization days
	BG control time	Mean FBG	FBG	Mean BG		
CSII group	0.201	0.509	0.649	0.968	0.097	0.100
MDI group	0.318	1.278	0.968	1.0233	0.182	0.225
Total mean	0.164	0.683	0.674	0.937	0.101	0.214

**Table 8:** Analysis of slack variables in patients undergoing grade IV surgery.

Group	Preoperative		Postoperative day 1		Days of antibiotic use	Postoperative hospitalization days
	BG control time	Mean FBG	FBG	Mean BG		
CSII group	0.104	0.639	0.639	0.865	0.122	0.102
MDI group	0.237	1.284	0.802	0.928	0.168	0.184
Total mean	0.109	0.821	0.694	0.937	0.105	0.132

## Data Availability

The datasets used and/or analyzed during the current study are available from the corresponding authors on reasonable request.
